# Open transversus abdominis plane block and analgesic requirements in patients following right hemicolectomy

**DOI:** 10.1308/003588412X13171221589856

**Published:** 2012-07

**Authors:** RR Brady, NT Ventham, DM Roberts, C Graham, T Daniel

**Affiliations:** ^1^NHS Fife,UK; ^2^Wellcome Trust Clinical Research Facility, Edinburgh,UK

**Keywords:** Analgesia, Laparotomy, Colorectal cancer, Transversus abdominis plane block, Local anesthesia

## Abstract

**INTRODUCTION:**

Reducing exogenously administered opioids in the post-operative period is associated with early return of bowel function and decreased post-operative complication rates. We evaluated the effectiveness of a surgeon-delivered open transversus abdominis plane (TAP) block as a method to reduce post-operative opioid requirements, sedation and inpatient stay.

**METHODS:**

The patient cohort was identified from those who had undergone a right hemicolectomy for colonic cancer. Patients received either an open TAP block and post-operative patient controlled anaesthesia (PCA) (*n*=20) or were part of a control group who received subcutaneous local anaesthetic infiltration and PCA (*n*=16).

**RESULTS:**

PCA morphine use was reduced within the first 24 hours post-operatively in the TAP block group compared with controls (42.1mg vs 72.3mg, *p*=0.002). Sedation was also reduced significantly in the early post-operative period (*p*<0.04). There was a non-significant trend towards reduced length of stay in the intervention group (8.2 vs 8.73 days). There were no recorded complications attributable to the open TAP block.

**CONCLUSIONS:**

Open TAP blocks are safe and reduce post-operative opioid requirements and sedation after right hemicolectomies. They should be considered as part of a multimodal enhanced recovery approach to patients undergoing abdominal surgery via a transverse incision.

Despite increased use of laparoscopic colorectal surgery, a national audit of colorectal cancer in 2010 revealed 75% of colonic resections for cancer are completed by the open approach.^1^ The principles of enhanced recovery after surgery (ERAS) include early resumption of oral intake, early initiation of mobilisation, avoidance of routine bowel preparation, nasogastric tubes and drains, and optimisation of post-operative analgesia. This aims to improve patient satisfaction and allow early discharge from hospital.^2^ A surgical insult leads to an inflammatory, endocrine and metabolic stress response, mediated by, among others, afferent neural stimuli activating the autonomic nervous system.^3^ Uncontrolled post-operative pain is associated with respiratory complications, myocardial ischaemia, cognitive impairment and prolonged hospital stay.^3^

The transversus abdominis plane (TAP) technique, originally described by Rafi,^4^ involves injection of local anaesthetic in the plane between the internal oblique and transversus abdominis muscle layers, with the aim of anaesthetising the intercostal nerves supplying the abdominal wall. Initially, the block used surface landmarks of the triangle of Petit (latissimus dorsi posteriorly, external oblique superiorly, iliac crest inferiorly) and a double fascial ‘pop’ (loss of resistance) to guide placement of the local anaesthetic.^4^ More recently, ultrasonography has been used to guide the delivery of the injectate into the appropriate plane, thereby increasing the accuracy of the technique.^5–9^ A subcostal technique aimed at providing analgesia for upper abdominal operations has also been described.^8,9^

However, in some reports the safety of TAP blocks has been raised.^10^ There have also been reports of liver injury caused by needle damage.^11,12^ Theoretical concerns have also been discussed regarding the risk of femoral nerve palsy.^13,14^ Landmark techniques and utrasonography guided placement of TAP blocks may also be more difficult in obese patients.^4^

Recently, surgically administered TAP blocks have been described, allowing a more accurate placement.^15–17^ This involves the operating surgeon indentifying the anatomical layers under direct vision when closing the abdomen and placing the local anaesthetic accordingly. This has the advantage of avoiding inadvertent injection into the incorrect layer or damaging deeper structures.

Our study evaluated the efficacy of open surgically placed TAP blocks. Open or surgically placed TAP blocks have been described as adjuncts in plastic,^16^ gynaecological^15^ and also colorectal surgery through a midline incision.^17^ Owen *et al* described a technique of performing an open surgical TAP block in women undergoing Caesarean section under spinal anaesthesia.^15^ They found a significantly lower morphine requirement in those with a surgically placed TAP compared with no TAP block. Bharti *et al* performed a small randomised controlled trial by injecting either 40ml of 0.25% levobupivacaine or saline from inside the abdominal wall into the TAP plane for colorectal resections.^17^

The aim of our study was to detect whether a surgeon administered open TAP block was an effective adjunct in providing post-operative analgesia in patients undergoing colorectal resection. Specifically, we focused on the impact of TAP blocks on reducing post-operative patient controlled anaesthesia (PCA) morphine requirements as a surrogate marker of post-operative pain. Additionally, we investigated whether TAP blocks lead to a reduction in sedation levels and a shorter hospital stay.

## Methods

The patient cohort was identified from a prospectively gathered cancer database for all colonic cancer patients who underwent an open right hemicolectomy between 2006 and 2011 at Queen Margaret Hospital, NHS Fife. Strict inclusion criteria were designed to ensure that a homogenous cohort was obtained. Patients were excluded from analysis if they had not had their operation under one specific experienced colorectal surgeon (who was the primary operator in all cases), had any incision other than a right upper transverse incision, had their operation performed as a non-elective (ie emergency) procedure or had missing data/details or non-recoverable inpatient notes. Patients were also excluded if they had an alternative post-operative analgesia plan (epidural analgesia, ultrasonography guided TAP placement, local anaesthetic wound catheters and non-morphine PCA).

Patient demographic details (sex, age), operative details (primary operator, incision, indication, urgency, timing and dose of TAP block placement) and post-operative details were recorded. The total PCA morphine intake in the first 24 hours post-operatively and in the second 24 hours (24–48 hours) was recorded. In addition, excessive sedation in the immediate 48-hour post-operative period was determined as the number of times that the sedation score was >2 in a 24-hour period (Ramsay sedation scale; best eye opening response of the patient: 1 = spontaneously, 2 = to speech, 3 = to stimulation, 4 = no response; recorded on an hourly basis). The total inpatient post-operative stay was calculated from the inpatient’s notes.

### Operative methodology

All patients underwent an elective open right hemicolectomy performed by one experienced colorectal surgeon. All operations were carried out through an upper right transverse incision. In the TAP group, 20ml of 0.5% levobupivacaine was infiltrated into the right TAP under direct vision at the time of wound closure. In the control group, 20ml of 0.5% levobupivacaine was infiltrated into the subcuticular space prior to skin closure. Both groups had post-operative PCA morphine. Analgesia in both groups was supplemented by intravenous paracetamol 1g four times a day for the first 48 hours. Additional morphine boluses of 5mg could be administered by attending medical staff if pain was not controlled sufficiently with the standard patient administered 1mg bolus and 5-minute lock-out.

Data were recorded on a proforma prior to being transferred to an Excel® worksheet (Microsoft, Redmond, WA, US). Comparisons between groups were made using either a two-sample t-test or the Mann–Whitney U test as appropriate using Minitab® 15 (Minitab, Coventry, UK). The threshold for statistical significance was *p*<0.05.

## Results

A total of 74 patients were identified as suitable for the patient cohort. Of these, 38 were excluded (21 epidural anaesthesia, 1 fentanyl PCA, 1 ultrasonography placed TAP block, 3 wound catheters and 12 patients with missing data), leaving 36 patients for analysis. Of these, 16 (44%) were managed with PCA morphine and local anaesthetic skin infiltration, and 20 (56%) with PCA and a surgically placed open TAP block. There was no evidence of difference between the groups in terms of sex (50% vs 55% men respectively, *p*=0.77) or age (mean: 68.3 years [SD: 10.4 years] vs 70.6 years [SD: 12.3 years] respectively, *p*=0.55).

**Figure 1:**
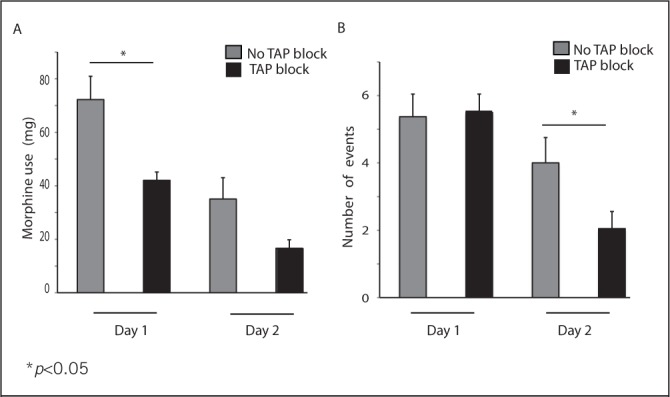
The effect of transversus abdominis plane (TAP) block on mean post-operative morphine requirements (A) and the mean number of excessive sedation events (B) over 24 hours

The amount of PCA morphine required by patients on post-operative days 1 and 2 is shown in Figure 1a. Comparing both groups, there was a significant difference in morphine consumption on day 1 (PCA + skin infiltration group mean usage was 30.2mg greater than the PCA + TAP group, 95% CI: 10.7–49.7mg, *p*=0.004). In the second 24 hours post-operatively, morphine use in those receiving PCA and a TAP block was half that of the PCA and skin infiltration group. However, this did not reach statistical significance (mean morphine use in PCA + TAP group: 16.65mg, mean use in PCA + skin infiltration group: 35.06mg, *p*=0.131).

* *p*<0.05

The number of episodes of excessive sedation is recorded in Figure 1b. There was a significant reduction on day 2 in the number of episodes of excessive sedation experienced by patients in the TAP block intervention group (difference: 1.95, 95% CI: 0.1–3.8, *p*=0.041). However, the difference in episodes of excessive sedation on day 1 between the two groups failed to reach statistical significance (difference: -0.1, 95% CI: -1.9–1.6, *p*=0.886). There was a marginal trend towards a shorter inpatient stay in the TAP intervention group versus the control group (mean: 8.2 vs 8.7 days).

## Discussion

In this study we found that a surgically administered TAP block significantly reduces the post-operative opioid requirements in the first 24 hours following an open right hemicolectomy. The morphine requirement in the TAP group was half of that in the control group during the second 24 hours but this failed to reach statistical significance. In addition, lower levels of excessive sedation were found in the second 24 hours in those patients who had received a surgically placed TAP block.

PCA provides analgesia that improves patient satisfaction.^3,18^ However, exogenously administered opioids reduce propulsive peristaltic contractions and increase the non-propulsive contractions, leading to side effects such as post-operative nausea and vomiting, and gastrointestinal paralysis.^19,20^ Consequently, reducing post-operative opioid administration could potentially reduce the occurrence of post-operative ileus and reduces morbidity, facilitating earlier discharge.^20^

TAP blocks have been recognised as playing an important role in effective multimodality post-operative analgesia.^21^ They have been shown to reduce opioid requirements in a Cochrane meta-analysis published in 2010^22^ although this was with eight relatively small heterogeneous studies. Anaesthesiologist-delivered TAP blocks have been found to reduce the morphine requirement and pain scores in Caesarean sections^7^ and open abdominal surgery^23^ in the context of randomised controlled trials albeit with relatively small patient numbers. In the context of laparoscopic colorectal resections, TAP blocks have been shown to reduce opioid requirements,^24^ the time to normal diet and time until hospital discharge when compared with opioid PCA in non-randomised trials.^25^

Our results echoed a randomised study comparing landmark technique TAP block and PCA versus standard PCA in patients undergoing a bowel resection through a midline laparotomy scar.^23^ Patients receiving a TAP block used 21.9mg of morphine compared with 80.44mg in those without a TAP block in the first 24 hours. This is comparable with the 42.05mg in those with a TAP block compared with 72.25mg without in our study. A possible explanation for the higher morphine requirement in our study was the unilateral administration of TAP block compared with the bilateral blocks used by McDonnell *et al*.^23^

The importance of this study lies in demonstrating the efficacy of open surgically placed TAP blocks, a developing technique. Currently, no studies have shown an open TAP block to be effective in reducing the morphine requirement in those undergoing a colonic resection via an upper abdominal transverse incision. However, these results should be interpreted with some caution due to the retrospective design of the study and the relatively small patient numbers. Nevertheless, the information presented here will allow future studies to be sufficiently powered to detect differences that are clinically relevant.

Some authors have suggested that TAP blocks as administered though the triangle of Petit may not be effective for upper abdominal procedures as there may not be an adequate sensory block of the lower six thoracic nerves.^26^ In a paper in which ultrasonography guided TAP blocks were used in laparoscopic colorectal resections, with specimen extraction and colonic anastomosis through a right upper quadrant incision, no significant statistical difference in morphine requirements in those with and without ultrasonography guided TAP block was demonstrated.^24^ Subcostal TAP blocks have also been described as a method of analgesia for upper abdominal operations, not extending below the T10 dermatome or more lateral than the anterior axillary line.^8,9^ Surgically placed TAP blocks negate these limitations as the block is placed locally rather than for an ‘upper’ (subcostal infiltration) or ‘lower’ (triangle of Petit infiltration) abdominal incision.

We experienced no complications with surgically administered TAP blocks. There have been several reports of inadvertent liver injury related to both traditional landmark and ultrasonography guided TAP blocks.^11,12^ In patients undergoing a right upper quadrant incision for an open hemicolectomy, surgeon administered open TAP blocks may avoid this complication. Similarly, intraperitoneal infiltration may occur in obese patients and in those with reduced muscle tone, even with ultrasonography guidance.^27^ One of the major strengths of surgically administered TAP blocks in open surgery is the ability to infiltrate the correct anatomical layer under direct vision, avoiding potential complications of transabdominal peritoneal puncture.

## Conclusions

Surgically administered TAP blocks significantly reduce opioid analgesic requirements in the immediate post-operative period following open right hemicolectomy. Patients who received a surgically placed TAP block had significantly fewer episodes of excessive sedation in the post-operative period. We conclude that surgically administered TAP blocks are safe and should be considered as part of the multimodal management of patients undergoing open colorectal surgery.
